# Introduction of ultrasound-based living anatomy into the medical curriculum: a survey on medical students’ perceptions

**DOI:** 10.1186/s13089-021-00247-1

**Published:** 2021-12-04

**Authors:** Pelagia Kefala-Karli, Leandros Sassis, Marina Sassi, Constantinos Zervides

**Affiliations:** 1 School of Medicine, University of Nicosia, 21 Ilia Papakyriakou Street, 2414 Engomi, Nicosia Cyprus; 2 Children’s Hospital “Agia Sofia”, Thivon Ave. and Papadiamantopoulou Street, 11527 Athens, Greece; 3 Department of Medical Physics and Clinical Engineering, Mediterranean Hospital of Cyprus, 9 Stygos Str., 3117 Limassol, Cyprus

**Keywords:** Ultrasound teaching, Medical education, Living anatomy, Medical students

## Abstract

**Background:**

Traditional anatomy teaching methods are based on the models and cadaveric dissections, providing fixed views of the anatomical structures. However, in the last few years, the emerging concept of ultrasound-based teaching in anatomy has started to gain ground among medical curricula. This study aims to evaluate the integration of ultrasound as an adjunct tool to traditional anatomy teaching methods and explore students’ perceptions of whether ultrasound-based teaching enhances their interest and knowledge of anatomy. A cross-sectional study was carried out among the students of the 6-year undergraduate entry (MD) and 4-year graduate entry (MBBS) program of the University of Nicosia. A questionnaire was distributed to them after the delivery of several twenty minutes ultrasound sessions by an expert in the field during anatomy practicals. The data were analyzed utilizing SPSS software, and the statistical significance was determined as *p* value < 0.05.

**Results:**

107 MD and 42 MBBS students completed the questionnaire. Both groups agreed that their ultrasound-based learning experience was good or excellent (79.4% MD students; 92.9% MBBS students), that it enhanced their knowledge of anatomy (68.2% MD students; 90.5% MBBS students) and boosted their confidence regarding their examination skills practice (69.2% MD students; 85.7% MBBS students). Although most students desired more time allocated to the ultrasound station (72% MD students; 85.7% MBBS students), they believed that ultrasound-based teaching is a necessary adjunct to the traditional teaching methods of anatomy (89.7% MD students; 92.9% MBBS students).

**Conclusions:**

Overall, MBBS students were more confident about the benefits of ultrasound-based teaching. Most of the students agreed that cross-sectional sessions of traditional teaching and ultrasound-based teaching strengthened their knowledge of anatomy and enhanced their confidence concerning their clinical examination skills. Medical schools should embrace the advantages that ultrasound-based teaching offers in order future doctors to be qualified to utilize ultrasound for procedural and diagnostical purposes.

**Supplementary Information:**

The online version contains supplementary material available at 10.1186/s13089-021-00247-1.

## Background

Anatomy is considered to be an integral part of undergraduate medical curricula. Traditionally, the teaching of human anatomy is based on the anatomical models, prosections, and cadavers. Nevertheless, nowadays, there is an ongoing debate concerning the means of delivery of anatomy sessions, where a shift towards living anatomy is noted [1].

Ultrasound-based teaching is the cornerstone of living anatomy and offers medical students the opportunity to observe in real time the movement of anatomical structures, understand physiology and hemodynamics, differentiate between normal and pathological variants, and to appreciate the relationship between surface and deep anatomy [2, 3]. Also, by utilizing ultrasound as a teaching anatomy method, medical students will familiarize themselves with the equipment and interpretation of ultrasound images. These skills are essential during their early clinical practice [2].

Principally, in preclinical courses, such as anatomy, emphasis is placed on memorization rather than understanding. Therefore, the exploitation of ultrasound as a teaching method in anatomy sessions in undergraduate medical curricula is currently being evaluated. During the last decade, studies [2, 4–13] showed that ultrasound is a beneficial educational tool in which students reacted positively to its incorporation in their anatomy sessions. Indeed, medical students agreed that ultrasound-based teaching enhances their self-confidence about identifying anatomical structures and improves their overall anatomical knowledge. Consequently, the fact that sonography training was initiated during the anatomy course in the medical schools, which have already incorporated a vertical ultrasound curriculum, was expected [14]. The recent research demonstrated that the anatomy faculty also reported favorable views in regard to the beneficial influence of ultrasound in anatomy education, and they acknowledged that sonography strengthens the teaching of anatomical concepts [15]. However, according to a recent study, only a few universities across Europe exploit ultrasound-based teaching in their anatomy sessions [16]. The anatomy practicals of most European medical schools are based on the cadavers and models and only a few medical schools pioneered and integrated ultrasound-based teaching in their curricula [16–18]; on the contrary, this step was carried out in Northern American medical schools during the last decade [19, 20].

Although the literature has shown that ultrasound integration in anatomy curricula was beneficial for medical students, conclusive outcomes are limited. In 2019, the Ultrasound Institute of the Medical School of the University of Nicosia investigated the upcoming, intriguing concept of integration of ultrasound in medical education, and based on the encouraging outcomes of the study, an ultrasound curriculum was integrated into the anatomy course [18]. Thus, the students of the 6-year undergraduate entry (MD) and 4-year graduate entry (MBBS) program were introduced to a structured ultrasound-based teaching for the first time during their studies. This could be the dawn of integrating ultrasound-based teaching into a broader context of medical curriculum; a step that will offer to the future doctors necessary skills for their career. This research aims to narrow the current literature gap by assessing whether ultrasound as an adjunct tool to traditional anatomy teaching methods helps medical students improve their knowledge of anatomy based on their perception.

## Methods

A cross-sectional study was conducted among second-year medical students of the 6-year program of the University of Nicosia Medical School (MD-degree program) and of the 4-year program of the St. George’s University of London, which is delivered by the University of Nicosia Medical School (MBBS-degree program). The purpose of this study was to evaluate the integration of ultrasound-based teaching in anatomy course and assess whether it is beneficial on improving the students’ anatomy knowledge. A secondary aim of this study was to illuminate whether the students of the more traditional MD program had different perception regarding the introduction of ultrasound-based teaching in anatomy from the students of the innovative MBBS program. The root of the debate stands to the fact that anatomy course in the MD program is mainly based on the cadavers and lectures, whereas the course in the MBBS program is more adaptable to the modern expectations; the learning experience is supported by problem-based learning sessions and diagnostic imaging is integrated earlier and in more aspects of the curriculum.

A questionnaire (Additional file 1: Students' Questionnaire; Appendix I) was distributed to the students of the MD program after four anatomy practicals and the students of the MBBS program after two anatomy practicals. In both programs ultrasound teaching was delivered in twenty minutes’ small-group training sessions by an expert in this field utilizing a Mindray DC-40 diagnostic ultrasound system. Specifically, the ultrasound training sessions took place along with the traditional gross dissection anatomy practicals. The students formed small groups of 4–6 people and rotated through 20 min ultrasound practical sessions in a room adjacent to the main anatomy lab. In each group one student volunteered to be the model while the rest of the group had hands-on experience by locating and obtaining focused images of the relevant anatomic structures that were earlier demonstrated on cadavers. The topics covered during the ultrasound-based teaching in each practical session are demonstrated in Table 1. The selection of these topics for each program was based on the theoretical anatomic content delivered to the students to achieve a parallel presentation of anatomic structures in theory, and in cadaveric and ultrasound demonstration. The study sample included a total of 161 medical students who attend lectures and laboratories (labs) by physical presence; 119 were registered in the MD program, and 42 were registered in the MBBS program.

**Table 1:** Ultrasound-based teaching sessions

Anatomy practicals for MD degree	
Practical session 1	Introduction to the ultrasound equipment
Practical session 2	Identification of the four chambers of the heart using ultrasound and A.I. (APEX 4 chamber view)
Practical session 3	Anatomy of the heart and vessels using ultrasound. Identification of the heart valves using ultrasound (Parasternal Long and Short Axis views)
Practical session 4	Interpretation of a basic ultrasound lungs examination
Anatomy practicals for MBBS degree	
Practical session 1	Introduction to the ultrasound equipment—Carotid arteries (Neck vasculature ultrasound)
Practical session 2	Interpretation of a basic ultrasound lungs examination

The questionnaire was designed in congruence with the previous studies [2, 6, 7, 11, 15, 20–22] and with the contribution of an expert in the field to ensure validity before it was given to the students to fill it out in a printed form. The questionnaire was composed of a demographic part, which included three multiple-choice items, and a part of twelve items. In the latter, students were asked to rate their experience of ultrasound-based teaching in anatomy practicals and evaluate whether the utilization of ultrasound demonstrations during these practicals was valuable for enhancing their knowledge of anatomy. Out of these twelve items, two were multiple response questions, one was multiple choice question, two were five-point Likert scales ranging from very poor to excellent, and seven were five-point Likert scales ranging from strongly disagree to strongly agree.

The results were statistically analyzed utilizing the *χ*^2^ test to compare binary or nominal variables and the Mann–Whitey *U* test to compare continuous or categorical variables. Statistical significance was determined as *p* value < 0.05 (5% Significance Level). All analyses were performed using the statistical software SPSS [23].

Participation in this survey was voluntary and complete anonymity was assured. This study was approved by the Cyprus National Bioethics Committee (CNBC).

## Results

One hundred seven second-year students registered in the MD program (MD students) and 42 first-year students registered in the MBBS program (MBBS students) completed the questionnaire; a total of 149 responses were received. The response rate was 89.9% for the MD students, and 100% for the MBBS students with an average response rate of 92.5%. The vast majority of the MD students were aged 18–20 years (72.9%), whereas a significant percentage of the MBBS students were aged 21–25 years (61.9%). Regarding the gender of the participants, 66 were males (45 MD students; 21 MBBS students), and 83 were females (62 MD students; 21 MBBS students). A detailed breakdown of the participants’ demographic characteristics is demonstrated in Table 2.

**Table 2:** Students’ demographic characteristics

	MBBS students (%)	MD students (%)	Total (%)	*p* value
Gender				
Male	21 (50)	45 (42.1)	66 (44.3)	0.464
Female	21 (50)	62 (57.9)	83 (55.7)	
Age group				
18–20	3 (7.1)	78 (72.9)	81 (54.5)	** < 0.001**
21–25	26 (61.9)	25 (23.4)	51 (34.2)	
26–30	11 (26.2)	4 (3.7)	15 (10.1)	
30 +	2 (4.8)	0 (0)	2 (1.3)	

Bold fonts indicate statistical significant values

Concerning the students’ evaluation of their ultrasound-based learning experience during the anatomy lab, the majority of the responders stated that it was good or excellent (79.4% MD students; 92.9% MBBS students) and that it was beneficial for their learning improvement of anatomy (68.2% MD students; 90.5% MBBS students). A statistically significant difference was noted between the MD and MBBS students (*p* value < 0.001).

A strong agreement or agreement was noticed among all of the participants regarding the statement that ultrasound-based teaching effectively demonstrated anatomy on a living human body (90.7% MD students; 95.2% MBBS students), helping them identifying organs or structures (65.4% MD students; 90.5% MBBS students) and reinforcing the knowledge of the anatomical structures which had been already presented in other anatomical sources (72% MD students; 95.2% MBBS students). For the above results, there were statistically significant differences between the MD and MBBS students (*p* value < 0.001).

Of all participants, 89.7% of MD students and 92.9% of MBBS students strongly agreed or agreed that ultrasound-based teaching is a necessary adjunct to the traditional teaching methods during the anatomy lab, making the session more interesting (83.2% MD students; 92.9% MBBS students), and providing them with more confidence in their examination skills and future medical practice (69.2% MD students; 85.7% MBBS students). A statistically significant difference was observed between MD and MBBS students (*p* value < 0.001). Indeed, the vast majority of the responders shared the belief that a cross-sectional session combining traditional teaching methods and ultrasound-based teaching would be more beneficial for their anatomy knowledge (89.7% MD students; 92.9% MBBS students).

Although nearly 3/4 of all the participants identified the limited time of allocation in ultrasound station as the most significant drawback (72% MD students; 85.7% MBBS students; *p* value = 0.021), 59.5% of the MBBS students believed that it is feasible to integrate ultrasound-based teaching in the current curriculum. However, 50.5% of the MD students had the view that ultrasound-based teaching could be incorporated into the clinical skills courses.

The statistically significant differences which were observed between the MD and MBBS students are demonstrated in Tables 3, 4, 5. In any case that a statically significant difference was noticed among the MD and MBBS students, the latter tended to be more positive and confident regarding ultrasound-based teaching in anatomy.

**Table 3:** Students’ responses regarding their ultrasound-based teaching experience

	MBBS students (%)	MD students (%)	Total (%)	*p*-value
Which of the following do you believe is more beneficial to your anatomy knowledge?				
Traditional teaching methods	3 (7.1)	8 (7.5)	11 (7.4)	0.545
Ultrasound-based teaching	0 (0)	3 (2.8)	3 (2)	
Cross-sectional session combining traditional teaching methods and ultrasound-based	39 (92.9)	96 (89.7)	135 (90.6)	
Which of the following is the most important drawback regarding ultrasound-based based teaching that you identified?				
Not enough time allocated in ultrasound station	36 (85.7)	77 (72)	113 (75.8)	**0.021**
Lack of ultrasound equipment	1 (2.4)	10 (9.3)	11 (7.4)	0.16
Difficulty of understanding ultrasound	5 (11.9)	22 (20.6)	27 (18.1)	0.261
Lack of faculty	1 (2.4)	2 (4.7)	3 (2)	0.81
None	0 (0)	5 (4.7)	5 (3.4)	0.164
Do you believe that it is feasible to integrate ultrasound:				
In the current anatomy curriculum	25 (59.5)	45 (42.1)	70 (47)	**0.027**
In the clinical skills courses	18 (42.9)	54 (50.5)	72 (48.3)	0.522
In other basic science courses (physiology, pathology)	9 (21.4)	16 (15)	25 (16.8)	0.278
As a separate course	16 (38.1)	20 (18.7)	36 (24.2)	**0.008**
Not at all	0 (0)	0 (0)	0 (0)	–

Bold fonts indicate statistical significant values

**Table 4:** Students’ evaluation of ultrasound-based teaching

	1 (%)	2 (%)	3 (%)	4 (%)	5 (%)	Mean Likert score (CI)	*p*-value
Ultrasound-based teaching in the course of anatomy lab							
MBBS students	1 (2.4)	1 (2.4)	0 (0)	5 (11.9)	34 (81)	4.75 (4.584–4.952)	**< 0.001**
MD students	0 (0)	4 (3.7)	18 (16.8)	50 (46.7)	35 (32.7)	4.085 (3.330–4.240)	
Total	0 (0)	5 (3.4)	19 (12.8)	55 (36.9)	69 (46.3)	4.267 (4.134–4.401)	
Your learning improvement of anatomy due to ultrasound							
MBBS students	1 (2.4)	2 (4.8)	0 (0)	13 (31)	25 (59.5)	4.5 (4.271–4.729)	** 0.001**
MD students	0 (0)	8 (7.5)	26 (24.3)	49 (45.8)	24 (22.4)	3.830 (3.663–3.997)	
Total	0 (0)	9 (6)	28 (18.8)	62 (41.6)	49 (32.9)	4.014 (3.870–4.157)	

Bold fonts indicate statistical significant values

**Table 5:** Students’ perception of whether ultrasound-based teaching enhanced their anatomy knowledge

	1 (%)	2 (%)	3 (%)	4(%)	5 (%)	Mean Likert score (CI)	*p*-value
Ultrasound demonstration is a necessary adjunct to traditional teaching methods during anatomy lab							
MBBS students	0 (0)	0 (0)	2 (4.8)	12 (28.6)	27 (64.3)	4.6 (4.411–4.789)	** < 0.001**
MD students	0 (0)	3 (2.8)	8 (7.5)	62 (57.9)	34 (31.8)	4.189 (4.056–4.322)	
Total	0 (0)	3 (2)	10 (6.7)	74 (49.7)	61 (40.9)	4.301 (4.189–4.414)	
Ultrasound-based teaching made anatomy lab more interesting							
MBBS students	0 (0)	0 (0)	2 (4.8)	6 (14.3)	33 (78.6)	4.75 (4.576–4.924)	** < 0.001**
MD students	0 (0)	7 (6.5)	11 (10.3)	47 (43.9)	42 (39.3)	4.160 (3.994–4.327)	
Total	0 (0)	7 (4.7)	13 (8.7)	53 (35.6)	75 (50.3)	4.322 (4.186–4.458)	
Ultrasound-based teaching helped me identifying organs and structures in the human body							
MBBS students	0 (0)	0 (0)	3 (7.1)	12 (28.6)	26 (61.9)	4.55 (4.346–4.754)	** < 0.001**
MD students	2 (1.9)	9 (8.4)	26 (24.3)	39 (36.4)	31 (29)	3.830 (3.636–4.025)	
Total	2 (1.3)	9 (6)	29 (19.5)	51 (34.2)	57 (38.3)	4.027 (3.868–4.187)	
Ultrasound helped me to reinforce my knowledge of the anatomical structures I have seen in other anatomical resources							
MBBS students	0 (0)	1 (2.4)	2 (4.8)	12 (28.6)	26 (61.9)	4.525 (4.296–4.754)	** < 0.001**
MD students	3 (2.8)	6 (5.6)	21 (19.6)	43 (40.2)	34 (31.8)	3.934 (3.741–4.126)	
Total	3 (2)	7 (4.7)	23 (15.4)	55 (36.9)	60 (40.3)	4.096 (3.938–4.254)	
Ultrasound imaging on a living human effectively demonstrated important anatomy							
MBBS students	0 (0)	0 (0)	1 (2.4)	8 (19)	32 (76.2)	4.75 (4.592–4.908)	** < 0.001**
MD students	0 (0)	2 (1.9)	8 (7.5)	55 (51.4)	42 (39.3)	4.283 (4.151–4.415)	
Total	0 (0)	2 (1.3)	9 (6)	63 (42.3)	74 (49.7)	4.411 (4.301–4.521)	
Study anatomy in the living human body with ultrasound was more beneficial for my anatomy learning than studying anatomy in cadavers only							
MBBS students	0 (0)	0 (0)	3 (7.1)	10 (23.8)	28 (66.7)	4.60 (4.398–4.802)	** < 0.001**
MD students	3 (2.8)	26 (24.3)	24 (22.4)	32 (29.9)	22 (20.6)	3.406 (3.184–3.628)	
Total	3 (2)	26 (17.4)	27 (18.1)	42 (28.2)	50 (33.6)	3.733 (3.542–3.923)	
Ultrasound training during the anatomy lab gave me more confidence in my physical exam skills/future medical practice							
MBBS students	0 (0)	0 (0)	5 (11.9)	8 (19)	28 (66.7)	4.550 (4.322–4.779)	**< 0.001**
MD students	1 (0.9)	9 (8.4)	23 (21.5)	42 (39.3)	32 (29.9)	3.877 (3.692–4.063)	
Total	1 (0.7)	9 (6)	28 (18.8)	50 (33.6)	60 (40.3)	4.062 (3.907–4.217)	

Bold fonts indicate statistical significant values

## Discussion

The utilization of ultrasound-based teaching as an adjunct tool to conventional teaching methods in anatomy course reinforces medical students' learning experience. Traditional anatomy demonstration methods such as dissection of cadavers or studying of plastinated specimens and atlases provide fixed views and show only an approximation of the living anatomy [15]. Unsurprisingly, the results of this study showed that pairing anatomy practical sessions with ultrasound significantly enhanced students’ knowledge of anatomy. Indeed, the majority of the students believed that the demonstration of anatomy concepts on a living human body helped them to identify organs and structures and concurrently strengthened their previous anatomy knowledge gained by other sources, such as prosections, models, and cross-sectional images. Hence, a significant increase in students’ self-reported interest towards anatomy practical sessions was expected (Mean Likert Score = 4.32; 95% CI = 4.19–4.46). Smith et al. (2018) [11] showed similar results when the ultrasound-based teaching was performed by a clinician and an anatomy teaching assistant during gross-anatomy sessions.

In this study’s cohort, a significant percentage of all students evaluated their ultrasound-based learning experience during anatomy sessions as good or excellent (Mean Likert Score = 4.27; 95% CI = 4.13–4.40). Indeed, both MD and MBBS students had the perspective that ultrasound-based teaching has an essential role in anatomy sessions and agreed that the cross-sectional sessions that combine traditional teaching methods and ultrasound-based teaching were more beneficial to their knowledge of anatomy. Indeed, systematic reviews [10, 13] showed that most of the students had a favorable perspective on the utilization of ultrasound in anatomy sessions.

In this survey, the students of the MD program had a different opinion than the students of the MBBS program in regard to which part of the curriculum it is feasible to integrate ultrasound; the majority of the latter (59.5%) thought that ultrasound could be incorporated in the current anatomy curriculum whereas nearly half of the MD students (50.5%) believed that ultrasound could be delivered in the clinical skills courses. Although institutions of different countries utilize variable methods to deliver ultrasound-based teaching, the medical students’ perspectives converge on the belief that ultrasound is beneficial for their anatomy learning and enhances their clinical skills [12, 13]. This perspective is in accordance with this study’s results. In fact, the vast majority of the MD students (Mean Likert Score = 3.88; 95% CI = 3.69–4.06) and the MBBS students (Mean Likert Score = 4.55; 95% CI = 4.32–4.78) reported that ultrasound training during the anatomy lab provided them with more confidence regarding their physical examination skills/future medical practice.

It is noteworthy that only a few medical curricula around the world have integrated ultrasound in anatomy up to date despite the several benefits that ultrasound-based teaching offers to students. This discrepancy indicates the presence of obstacles concerning the incorporation of ultrasound in anatomy sessions. Indeed, Royer et al. (2016) [15] showed that a significant barrier is that most of the faculty (65%), who are involved with the anatomy sessions, are not proficient users of ultrasound. In this study, according to the majority of the students (72% MD students; 85.7% MBBS students), the limited time of allocation of each student group in the ultrasound station was the most notable drawback.

This survey’s results showed that students of the MBBS program tended to be more confident and expressed more positive views concerning the ultrasound-based teaching in anatomy labs when compared to the students of the MD program. This deviation among the students’ perspective may illustrate either the different teaching methods used between the MD program and the MBBS program or the different students’ background knowledge since MBBS students already hold a bachelor’s degree. This study’s results are in accordance with Marom’s and Tarrasch’s research which showed that students of the 6-year program favored anatomy textbooks and cadaveric dissections as teaching methods whereas students of the 4-year program preferred imaging-based teaching [24]. Although this deviation in perspective between the two groups of students, our study demonstrated that most of them agreed that anatomy practicals combining both traditional and ultrasound-based teaching would be more beneficial for their anatomy knowledge. The aforementioned results illuminate the future aim of modern anatomy teaching which will incorporate living anatomy in combination with traditional cadaveric dissection and textbooks, provided that the teaching quality of neither of the components will be downgraded.

A limitation of this study might be the introduction of selection bias, which could result from the small sample size. Also, due to the study’s nature, which is based on the self-reported data, information bias might be present. Moreover, only a few sessions were delivered to the students before they were asked to fill out the questionnaire. Therefore, an improvement of this study could be the evaluation of students’ perspectives on ultrasound-based teaching in the long term. Future studies should be focused on assessing students’ performance regarding the ultrasound material taught during anatomy sessions and the perceptions of anatomy faculty about this innovative approach of anatomy teaching.

## Conclusions

This study demonstrated that the majority of the students agreed that the integration of ultrasound in anatomy labs was an essential adjunct to the traditional teaching methods, which enhanced their knowledge of anatomical structures and boosted their confidence regarding their physical examination skills and future medical practice. According to this survey’s results, a coordinated effort should be made in order to achieve a widespread integration of ultrasound-based teaching in medical curricula. Thus, future physicians will be qualified with the appropriate knowledge regarding the proper use of ultrasound in the context of procedural and diagnostical purposes.

## Supplementary Information

**Additional file 1.** Students' Questionnaire; Appendix I.

## Data Availability

The data used and/or analysed during the current study are available from the corresponding author on reasonable request.
