# Supplementary material for: Introduction of ultrasound-based living anatomy into the medical curriculum: a survey on medical students’ perceptions
Source: Ultrasound J. 2021 Dec 4;13:47. doi: 10.1186/s13089-021-00247-1 (PMC8643372; doi:10.1186/s13089-021-00247-1)
Supplement: Supplementary file 1 — Additional file 1. Students' Questionnaire; Appendix I. [file 13089_2021_247_MOESM1_ESM.docx]

**Appendix I**

***Questionnaire***

1. **Please select your gender:**

- Male
- Female

1. **Please select your age group:**

- 18-20
- 21-25
- 26-30
- 30+

1. **You are:**

- Student in the 4-Year MBBS program
- Student in the 6-Year MD program

1. **On a scale of 1 to 5, how do you rate:**

|  | 1. Very poor | 2. Poor | 3. Fair | 4. Good | 5. Excellent |
| --- | --- | --- | --- | --- | --- |
| Ultrasound-based teaching in the course of anatomy lab. |  |  |  |  |  |
| Your learning improvement of anatomy due to ultrasound |  |  |  |  |  |

1. **Which of the following do you believe is more beneficial to your anatomy knowledge?**

- Traditional teaching methods
- Ultrasound-based teaching
- Cross-sectional session combining traditional teaching methods and ultrasound-based teaching

1. **How strongly do you agree or disagree with the following statements?**

|  | 1. Strongly disagree | 2. Disagree | 3. Undecided | 4.  Agree | 5. Strongly agree |
| --- | --- | --- | --- | --- | --- |
| Ultrasound demonstration is a necessary adjunct to traditional teaching methods during anatomy lab. |  |  |  |  |  |
| Ultrasound-based teaching made anatomy lab more interesting. |  |  |  |  |  |
| Ultrasound-based teaching helped me identifying organs (e.g., heart) and structures (e.g., mitral valve) in the human body. |  |  |  |  |  |
| Ultrasound helped me to reinforce my knowledge of the anatomical structures I have seen in other anatomical resources (prosections, models, cross-sectional images, etc.) |  |  |  |  |  |
| Ultrasound imaging on a living human effectively demonstrated important anatomy. |  |  |  |  |  |
| Study anatomy in the living human body with ultrasound was more beneficial for my anatomy learning than studying anatomy in cadavers only. |  |  |  |  |  |
| Ultrasound training during the anatomy lab gave me more confidence in my physical exam skills/future medical practice. |  |  |  |  |  |

1. **Which of the following is the most important drawback regarding ultrasound-based teaching that you identified?**

- Not enough time allocated in ultrasound station
- Lack of ultrasound equipment
- Difficulty of understanding ultrasound
- Lack of faculty
- None

1. **Do you believe that it is feasible to integrate ultrasound:**

- In the current anatomy curriculum
- In the clinical skills courses
- In other basic science courses (physiology, pathology)
- As a separate course
- Not at all
